# Five novel glucose-6-phosphate dehydrogenase deficiency haplotypes correlating with disease severity

**DOI:** 10.1186/1479-5876-10-199

**Published:** 2012-09-24

**Authors:** Ashraf Dallol, Huda Banni, Mamdooh A Gari, Mohammed H Al-Qahtani, Adel M Abuzenadeh, Fatin Al-Sayes, Adeel G Chaudhary, Jeffrey Bidwell, Wael Kafienah

**Affiliations:** 1 School of Cellular and Molecular Medicine, Medical Sciences Building, University of Bristol, University Walk, Bristol, BS8 1TD, UK; 2 Center of Excellence in Genomic Medicine Research, King Abdulaziz University, Jeddah, Saudi Arabia; 3 Department of Hematology, Faculty of Medicine, King Abdulaziz University, Jeddah, Saudi Arabia

**Keywords:** Glucose-6-phosphate dehydrogenase, Haemolytic anaemia, DNA sequencing, Haplotype, PHASE reconstruction, Linkage analysis, Gene mutation

## Abstract

**Background:**

Glucose-6-phosphate dehydrogenase (G6PD, EC 1.1.1.49) deficiency is caused by one or more mutations in the *G6PD* gene on chromosome X. An association between enzyme levels and gene haplotypes remains to be established.

**Methods:**

In this study, we determined G6PD enzyme levels and sequenced the coding region, including the intron-exon boundaries, in a group of individuals (163 males and 86 females) who were referred to the clinic with suspected G6PD deficiency. The sequence data were analysed by physical linkage analysis and PHASE haplotype reconstruction.

**Results:**

All previously reported G6PD missense changes, including the AURES, MEDITERRANEAN, A-, SIBARI, VIANGCHAN and ANANT, were identified in our cohort. The AURES mutation (p.Ile48Thr) was the most common variant in the cohort (30% in males patients) followed by the Mediterranean variant (p.Ser188Phe) detectable in 17.79% in male patients. Variant forms of the A- mutation (p.Val68Met, p.Asn126Asp or a combination of both) were detectable in 15.33% of the male patients. However, unique to this study, several of such mutations co-existed in the same patient as shown by physical linkage in males or PHASE haplotype reconstruction in females. Based on 6 non-synonymous variants of G6PD, 13 different haplotypes (13 in males, 8 in females) were identified. Five of these were previously unreported (Jeddah A, B, C, D and E) and were defined by previously unreported combinations of extant mutations where patients harbouring these haplotypes exhibited severe G6PD deficiency.

**Conclusions:**

Our findings will help design a focused population screening approach and provide better management for G6PD deficiency patients.

## Background

The *G6PD* gene encodes the enzyme glucose-6-phosphate dehydrogenase (G6PD, EC 1.1.1.49). The enzyme is involved in the normal processing of carbohydrates and plays a critical role in red blood cells. It is responsible for the first step in the pentose phosphate cycle, a pathway that converts glucose to ribose-5-phosphate, which is the building block of purines and pyrimidines. G6PD catalyses the production of NADPH, which plays a major role in protecting cells from potentially harmful reactive oxygen species.

G6PD deficiency is the most common human metabolic inborn error affecting more than 400 million people worldwide [1], with the highest frequency observed in Africa, Asia, the Mediterranean and the Middle-East [2-4]. G6PD deficiency is caused by one or more mutations in the *G6PD* gene on chromosome X, which lead to functional variants of the protein resulting in different biochemical and clinical phenotypes. The most common clinical manifestations are neonatal jaundice and acute haemolytic anaemia, which in most patients is triggered by an exogenous agent [2]. In some cases, the neonatal jaundice is severe enough to cause death or permanent neurological damage. In a proportion of cases, these manifestations may be life-threatening but fortunately, apart from episodes of haemolytic anaemia, most G6PD-deficient individuals are usually asymptomatic. A very small proportion of G6PD-deficient individuals have chronic haemolytic anaemia which can be severe. Total loss of G6PD activity is fatal. Because the disorder has an X-linked recessive mode of inheritance, males are usually more severely affected than females, though homozygosity, compound heterozygosity, or skewed X-inactivation of affected chromosomes may produce symptoms in females [5].

There are over 190 recorded *G6PD* gene mutations: each has a characteristic distribution in different geographical regions and ethnic groups. The striking concordance between the areas where G6PD deficiency is common and those where *Plasmodium falciparum* malaria is endemic provides circumstantial evidence that G6PD deficiency confers resistance against malaria [4]. The global distribution of mutations correlates with historically recorded distributions of the disease [3,4,6-9].

To date, only one large scale systematic study of *G6PD* gene mutations associated with G6PD deficiency has been undertaken for the Saudi Arabian population, but this study did not describe pan-gene *G6PD* haplotyping nor sequencing of introns [10]. The aim of the present study was therefore to sequence the *G6PD* gene in suspected male and female G6PD-deficient patients from the Saudi population and to determine the patterns of mutation and polymorphism in *cis* (haplotypes) within the gene by physical linkage analysis in male patients, and by haplotype reconstruction in female patients. In addition, the correlation between specific *G6PD* haplotypes and the activity of G6PD was examined in all male patients, and in female patients who were homozygous or heterozygous for a given haplotype.

## Methods

### Patients

249 individuals with suspected G6PD deficiency were selected based on clinical observation (163 male, 86 female). The individuals enrolled originated mostly from the Western region of the Kingdom of Saudi Arabia. Members of this cohort ages ranged from newborn to 50 years. Blood samples were collected under conditions approved by the local Ethical Committee. All the individuals enrolled in this study were subjected to quantitative measurement of their G6PD enzyme levels as well as DNA extraction for G6PD mutational analysis.

### G6PD enzyme assay

Whole blood was used to quantitatively measure G6PD enzyme activity using a UDICHEM-310 spectrophotometer (United Diagnostics Industry, Damman, K.S.A). G6PD activity was determined for all samples according to WHO recommendations [11] using the UV/Kinetic method (United Diagnostics Industry G6PD quantitative kit 038–020, UDI, Dammam, K.S.A).

### G6PD gene PCR

Genomic DNA was extracted from whole blood and quantitated using standard methods (QIAamp DNA Blood Mini kit, Qiagen). PCR primers were designed using Primer 3.0 software (http://frodo.wi.mit.edu/primer3/). A *G6PD* reference sequence (NT 167198.1) was used to identify intron-exon boundaries. PCR primers were designed to amplify exons including associated intron-exon boundaries for the entire coding region. The primer length was typically 18–22 nucleotides with a GC ratio of about 50%. The specificity of the PCR primers was ascertained by performing homology searches using the NCBI BLAST tool. Oligonucleotides were synthesized by MWG Operon (Eurofins, Germany). Sequences of the PCR primers are shown in Table 1. Touchdown PCR reactions contained genomic DNA (100 ng), PCR forward primer (10pmol), PCR reverse primer (10pmol), dNTPs (10 mM), 0.5U Hot Start *Taq* DNA polymerase (Qiagen, 5U/μL), 10 X PCR buffer (Qiagen) containing 1.5 mM MgCl_2_ in a total volume of 25 μl. Samples were initially denatured at 95°C for 15 min. DNA amplification was performed using 30 cycles of denaturation at 95°C for 30 sec, initial annealing starting at 70°C for 30 sec, and extension at 72°C for 30 sec. The annealing temperature was reduced by 0.5°C at each subsequent cycle. Another 30 cycles of fixed annealing temperature PCR was performed, starting by denaturation at 95°C for 30 sec, annealing at 54°C for 30 sec and extension at 72°C for 30 sec. A final extension step at 72°C for 5 min was performed to allow the newly synthesized fragments to complete replication. All PCRs were performed using a Bio-Rad DNA Thermal Cycler (Bio-Rad, USA).

**Table 1:** **PCR primer sequences,*G6PD*gene fragments amplified and PCR product sizes**

**Frag-ment**	**Exon(s)**	**Forward primer sequence (5’-3’)**	**Reverse primer sequence (5’-3’)**	**PCR product size**
1	1,2	**CAATAGGGCCGGCTTGAC**	**TGCAACAATTAGTTGGAAAAGC**	1376bp
2	3,4,5	**TGTCCCCAGCCACTTCTAAC**	**CTCATAGAGTGGTGGGAGCA**	1125bp
3	6,7	**AGGGGTTCAAGGGGGTAAC**	**TGCAGGGTGACTGGCTCT**	563bp
4	8	**ACAGGGCGGGGAGCTAAG**	**GTGCCTCGTCACAGATGG**	202bp
5	9,10	**CCTGAGGGCTGCACATCT**	**GTGTCTTGCTGATGCCACTG**	741bp
6	11,12,13	**TGGCATCAGCAAGACACTCT**	**GACAAGGAAGTGGGTCCTCA**	1151bp

### DNA sequencing

PCR products were ethanol-precipitated and subjected to DNA sequencing using a BigDye Terminator v1.1/v3.1 Cycle Sequencing Kit (Applied Biosystems), and an Applied Biosystems automated DNA sequencer (ABI PRISM Genetic analyzer 3130, Hitachi).

### Genotype and haplotype analysis

Patient genotypes were assigned using BLAST alignment with the *G6PD* reference genomic DNA sequence NT_167198. All sequences matched the reference genomic sequence except for mutation or polymorphism sites at the loci shown in Table 2. Haplotypes in male patients were evident since males are hemizygous for the X chromosome. Haplotypes in female patients were reconstructed using maximum likelihood analysis (PHASE version 2.1) [12,13] with allowance for recombination and decay of linkage disequilibrium with distance. Linkage disequilibrium between adjacent pairs of mutant or polymorphic loci was determined by analysis of the Lewontin's |D'| linkage disequilibrium coefficient, using Haploview [14]. Unpaired t test is used for analysing the effect of G6PD mutations on its enzyme activity.

**Table 2:** Mutations and polymorphisms identified in patients

Locus*	**Exon /intron number**	Nucleotide number**	**Nucleotide mutation**	**Amino acid number and substitution**	**Designation**	**Frequency**		
						**F (N)**		**M (N)**
						**Hom.**	**Het.**	**Hemi.**
0	Exon 3	153417565	T>C	p.Ile48Thr	AURES	13	20	58
						(86)	(86)	(163)
1	Exon 4	153417411	G>A	p.Val68Met	A-	4	8	20
						(86)	(86)	(163)
2	Exon 5	153416686	A>G	p.Asn126Asp	A-	0	8	23
						(86)	(86)	(163)
3	Exon 6	153415828	C>T	p.Ser188Phe	MEDITERANEAN	9	20	36
						(86)	(86)	(163)
4	Exon 6	153415757	A>G	p.Met212Val	SIBARI	0	0	2
						(86)	(86)	(163)
5	Exon 9	153414531	G>A	p.Val291Met	VIANGCHAN	0	0	1
						(86)	(86)	(163)
6	Exon 9	153414434	T>C	p.Leu323Pro	A-	0	1	0
						(86)	(86)	(163)
7	Exon 11	153413848	C>T	p.Tyr437Tyr	-	14	18	51
						(61)	(61)	(102)
8	Intron	153413702	T>C	-	-	32	18	77
	11					(61)	(61)	(102)
9	Exon 12	153413666	G>A	p.Arg463His	ANANT	1	0	3
						(61)	(61)	(102)
10	Exon 12	153413623	C>T	p.Pro477Pro	-	0	1	1
						(61)	(61)	(102)
11	Intron	153413340	-/GGA	-	-	0	2	1
	13					(61)	(61)	(102)
12	Intron	153413052	A>G	-	-	1	3	1
	13					(61)	(61)	(102)

Footnote to Table 2: *, locus designation used throughout this communication and for the linkage disequilibrium analysis locus codes. ** Nucleotide numbers are from G6PD reference sequence (UCSC Genome Browser, February 2006 build).

## Results

Mutations and polymorphisms identified in the patient cohort, singly or in combination, are listed in Table 2. Mutations were subsequently identified as components of *G6PD* haplotypes by physical linkage or PHASE analysis as described. Comparisons of 6-locus *G6PD* haplotyping for both male and female patients are shown in Table 3. 6-locus *G6PD* haplotypes were identifiable directly because males are hemizygous for the X chromosome and therefore genotypes at each *G6PD* locus are in physical linkage.

**Table 3:** 6-locus haplotype PHASE reconstructions

**6-locus haplotype ID**	**Amino acid residue**						Females (N)*	**Males (N)**	
	**48**	**68**	**126**	**188**	**212**	**291**			
	**Locus 0 Exon 3**	**Locus 1 Exon 4**	**Locus 2 Exon 5**	**Locus 3 Exon 6**	**Locus 4 Exon 6**	**Locus 5 exon 9**			
1	T	G	A	C	A	G	76	43	NORMAL
2	**C**	G	A	C	A	G	45	49	AURES
3	T	G	A	**T**	A	G	34	29	MEDITERANEAN
4	T	G	A	C	**G**	G	0	2	SIBARI
5	T	G	A	C	A	**A**	0	1	VIANGCHAN
6	T	**A**	**G**	C	A	G	7	5	A-(1)
7	T	**A**	A	C	A	G	6	5	A-(2)
8	T	G	**G**	C	A	G	1	15	A-(3)
9	T	**A**	**G**	**T**	A	G	0	1	JA
10	T	**A**	A	**T**	A	G	2	2	JB
11	T	G	**G**	**T**	A	G	0	2	JC
12	**C**	**A**	A	C	A	G	1	7	JD
13	**C**	G	A	**T**	A	G	0	1	JE
							172	163	TOTAL

Footnote to Table 3. (a) Haplotypes identified by 6-locus haplotyping. Non-conservative mutated bases are shown in bold. Mutant loci are numbered from left to right and correspond to loci 0–5, as designated in Table 1. Totals represent haplotypes numbers, not patient numbers (except in males where these are identical). A-(1), A-(2) and A-(3) are variants of the A- phenotype [15]. (N)*=haplotype count.

6-locus haplotyping was performed for 163 patients and revealed 13 haplotypes. The selection of the loci was based upon extent of sequence coverage and the potential significance of the variation detected on protein function. One haplotype was 'normal', that is, possessed no amino acid substitutions in the region sequenced associated with G6PD deficiency: this was observed in 43 male patients (26.38%). In males, the three most common pathogenic 6-locus haplotypes observed in order of frequency were Aures, characterised by a single p.Ile48Thr mutation (49/163, 30.06%), Mediterranean, characterised by a single p.Ser188Phe mutation (29/164, 17.79%) and variant of A- designated A-(1–3) [15] characterised by a combination of p.Val68Met and p.Asn126Asp mutations (25/163, 15.33%). Other haplotypes were each present in less than 2% of male patients. However, 5 haplotypes were previously unreported (Jeddah A, B, C, D and E) and were all characterised by novel combinations of two or three non-conservative amino acid substitutions (Table 4). These novel haplotypes accounted for 13/163 (7.97%) of the male patients with Jeddah D (JD) being the most common novel haplotype as it was detectable in 7/163 male patients (4.29%).

**Table 4:** Novel haplotypes identified in Jeddah patients

Jeddah A	Val68Met+Asn126Asp+Ser188Phe
Jeddah B	Val68Met+Ser188Phe
Jeddah C	Asn126Asp+Ser188Phe
Jeddah D	Ile48Thr+Val68Met
Jeddah E	Ile48Thr+Ser188Phe

*G6PD* haplotypes were identified in female patients using PHASE haplotype reconstruction. 6-locus haplotyping was performed for 86 patients (172 haplotypes) and revealed 8 haplotypes. In females, the two most common pathogenic 6-locus haplotypes observed in order of frequency were Aures, (45/172, 26.16%) and Mediterranean (34/172, 19.76%). Other pathogenic haplotypes were each present with a haplotype frequency less than 5%. Only 2 of the novel Jeddah haplotypes were identified in the female patients (Jeddah B and Jeddah D).

**Figure 1:**
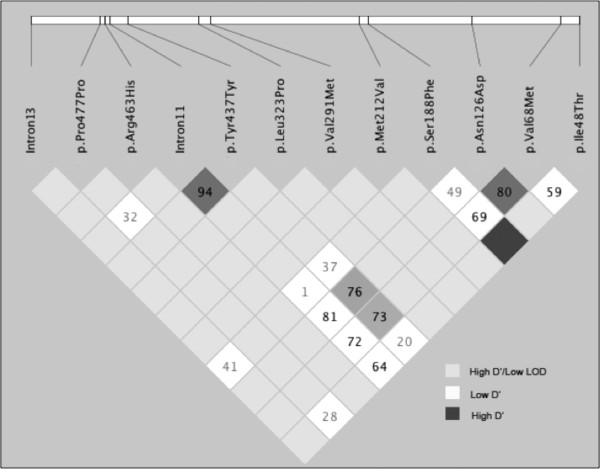
**Patterns of Linkage disequilibrium of the G6PD variations as demonstrated by Haploview output.** The D’ values are displayed and the shading of the boxes corresponding to D' values as shown in the legend.

Haplotype data permitted the analysis of linkage disequilibrium (LD) between pairs of adjacent mutant or polymorphic loci beyond the 6-loci used for haplotype analysis. As shown in Figure 1, the synonymous p.Tyr437Tyr and the T > C polymorphism in intron 11 exhibit a LOD score of 10.23 and D’ value of 0.947. Significant linkage disequilibrium exists between the p.Tyr437Tyr polymorphism and the p.Asn126Asp mutation (LOD = 2.53, D’ = 0.769) or the p.Val68Met mutation (LOD = 3.04, D’ = 0.734). Significant linkage disequilibrium is also detectable between p.Ser188Phe and p.Ile48Thr (LOD = 2.93, D’ = 1.0) and p.Asn126Asp with p.Val68Met (LOD = 21.03, D’ = 0.806), suggesting that several other haplotypes can be detected if the synonymous changes or intronic polymorphisms were to be included.

### Correlation between G6PD haplotype and G6PD enzyme levels

**Figure 2:**
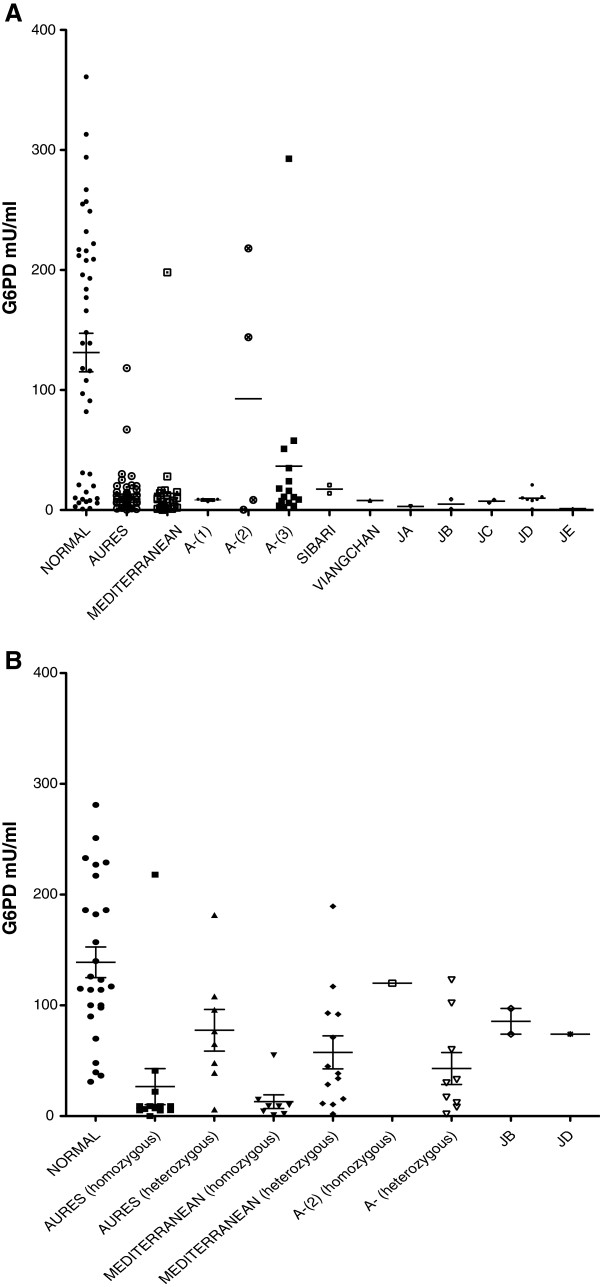
**Hapotype-phenotype correlation of the *G6PD *gene.** (**A**) correlation between *G6PD* haplotype and G6PD enzyme levels in male individuals with G6PD mutation. (**B**) correlation between *G6PD* haplotype and G6PD enzyme levels in females.

Figure 2 and Tables 5–6 illustrate the effect of different *G6PD* haplotypes on the level of G6PD expressed. The dominant effect on expression of a given haplotype could be assessed by analysing males who were hemizygous and females who were homozygous for that haplotype, since these individuals only possess the mutant haplotype. The effect on expression of a given haplotype in heterozygous females was also examined. In females who are carriers of a mutant *G6PD* haplotype, mosaic expression of G6PD on the affected X chromosome due to skewed X-inactivation can lead to clinical G6PD deficiency.

**Table 5:** G6PD enzyme values according to haplotype in male patients

**Males**	**Normal**	**Aures**	**Med.**	**A-(1)**	**A-(2)**	**A-(3)**	**Sibari**	**Viang.**	**JA**	**JB**	**JC**	**JD**	**JE**
N	43	49	29	5	4	15	2	1	1	2	2	7	1
Minimum	0.90	0.28	0.20	7.00	0.25	2.33	14.00	7.96	3.00	0.82	6.31	0.90	1.15
Maximum	361.00	118.20	198.00	9.00	218.00	292.70	20.84	7.96	3.00	9.00	8.43	21.00	1.15
Median	139.00	9.00	6.86	9.00	76.20	11.00	17.42	7.96	3.00	4.91	7.37	9.00	1.15
Mean	131.30	13.29	13.83	8.40	92.66	36.52	17.42	7.96	3.00	4.91	7.37	9.87	1.15
Std. Error	16.01	2.67	6.68	0.40	53.22	18.83	3.42	0.00	0.00	4.09	1.06	2.24	0.00
P-value		<0.0001	<0.0001	0.012	0.425	0.002	-	-	-	-	-	0.003	

P-values compared to normal G6PD levels.

**Table 6:** G6PD enzyme values according to haplotype in female patients

**Females**	**Normal**	**Aures (hom.)**	**Aures (het.)**	**Med. (hom.)**	**Med. (het.)**	**A-(2) (hom.)**	**A-(het.)**	**JB (het.)**	**JD (het.)**
N	26	13	8	8	13	1	9	2	1
Minimum	31	0.018	5.9	0.939	1.9	120	1.8	74	74
Maximum	281	218	181.6	55	189.4	120	123	97.17	74
Median	120	9	70.77	9.05	38.54	120	30	85.58	74
Mean	138.9	26.62	77.5	13.16	57.53	120	42.98	85.58	74
Std. Error	13.87	16.21	18.77	6.193	14.91	0	14.43	11.58	0
P-value		<0.0001	0.031	<0.0001	0.0008	-	0.0006	-	-

P-values compared to normal G6PD levels.

## Discussion

Our approach to a genetic analysis of G6PD deficiency in the Saudi population was to identify haplotypes of *G6PD* using a combination of DNA sequencing of exons 3, 4, 5, 6, 7, 9, 10, 11 and 12 and introns 11 and 13, utilising physical linkage to define *G6PD* haplotypes in male patients, and PHASE analysis to reconstruct haplotypes in female patients. Definition of haplotype phase in female patients who are compound heterozygotes (for mutations or polymorphisms) can be addressed successfully using PHASE reconstruction of haplotypes, when a suitably significant number of patients are available, as was the case in our study. Haplotype analysis addresses not only the distribution of pathogenic mutations, but their linkage in *cis* with other pathogenic mutations and polymorphisms, and thus permits linkage disequilibrium analysis for any given gene. In this communication, we report the results of a 6-locus haplotypic analysis of the *G6PD* gene in Saudi patients suffering from G6PD deficiency. 6-locus haplotyping identified 13 haplotypes in males (1 normal, 12 pathogenic) and 8 in females (1 normal, 7 pathogenic). Extending the haplotype analysis loci distal to exon 9 this resulted in only one further pathogenic mutation being identified (Anant, Arg463His). However it generates a significant amount of information about polymorphic variants within the gene and increased the total number of haplotypes observed. Analysis of pairwise linkage disequilibrium revealed significant linkage patterns between specific pairs of loci.

To date, only a few previously published reports of G6PD phenotype-genotype correlations have identified genotypes that represent combinations of more than one non-conservative mutation. Indeed, in a review by Beutler & Vulliamy [15-17] only 10 of 140 non-conservative amino acid mutations are combinations of more than one mutation. The present study has identified 5 novel, pathogenic *G6PD* haplotypes (Jeddah A through E), all of which are represented by combinations of 2 or 3 non-conservative amino acid substitutions (Table 2). Even allowing for the fact that these combinations are rare in the Saudi population and may be rare in other populations, this provides a significant insight into the cumulative mutation process in the *G6PD* gene.

We examined the effect of *G6PD* haplotype on levels of expression of G6PD in order to identify haplotypes which conferred the greatest clinical burden to patients. Our data supports the hypothesis that *G6PD* haplotypes representing two or more non-conservative amino acid mutations confer a greater reduction in G6PD expression than in haplotypes which only represent a single non-conservative amino acid mutation. This is perhaps unsurprising since the structure of the G6PD enzyme is more abnormal in the former cases.

Identification of the novel Jeddah A, B, C, D and E haplotypes could prove extremely important, since even carriers of two or more non-conservative amino acid mutations appear to have clinically significant G6PD deficiency.

The advantages of a haplotypic analysis of the *G6PD* gene is beyond doubt. We elected to pursue this approach to provide a wider viewpoint of *G6PD* genetics than a study focussed on the detection of single mutations. Detection of a given haplotype in males in our study provided confidence that identification of that haplotype in females represented confirmed linkage, not a computational artifact of the PHASE analysis.

Despite a significant effort in sequencing the *G6PD* gene in our patient cohort, ‘normal’ *G6PD* haplotypes were identified in 43 hemizygous male patients and in 26 female patients who possessed two apparently ‘normal’ X chromosomes. This may indicate the presence of a pathogenic mutation situated outside the regions covered by our sequencing strategy which may affect G6PD expression, but nonetheless our data is consistent with other studies [10] in which no *G6PD* mutations have been detected.

The predominant *G6PD* haplotypes in our patient cohort represent previously reported Aures (p.Ile48Thr) and Mediterranean (p.Ser188Phe) haplotypes. However, this report represents the first complete haplotypic analysis of the *G6PD* gene in patients from Saudi Arabia. We describe 5 previously unreported *G6PD* haplotypes which are characterised by novel combinations of extant mutations. These novel combinations pose interesting questions as to the existence of, and interplay between, putative mutation hotspots within the *G6PD* gene.

## Conclusions

Over 400 million people worldwide suffer from a deficiency of G6PD that results in mild to severe anaemia. The deficiency is caused by a range of inherited genetic mutations, but is most usually caused by a single mutation in the G6PD gene. We have discovered 5 new examples where more than one mutation exists in the same gene. These mutations have been seen singly in previous studies, but never in multiple combinations. The effect of the multiple mutations is to produce a more severe G6PD deficiency, which increases in severity according to the number of accumulated mutations. Females, who are normally only carriers of the disease, are generally unaffected or only show mild deficiency. However, our study suggests that females who possess multiple mutations are more G6PD-deficient than carriers of single mutations. This observation adds further complexity to G6PD deficiency testing as the severity of the disease may also be determined by the position of the mutation in the protein as some mutations may affect the G6PD functional domains. Our study demonstrates that during genetic testing, it is now important to test for multiple mutations, since these are associated with the severity of G6PD deficiency. Identification of multiple mutations in patients could therefore be used predictively to identify patients at risk of severe disease.

## Competing interests

Some of the findings in this work are being considered for patent application.

## Authors’ contributions

AD and HB performed research, collected data, analyzed and interpreted data, performed statistical analysis, and co-wrote the manuscript; MAG, MHA, AMA, FA and AGC supplied samples, interpreted data and contributed vital new reagents or analytical tools; WK and JB designed research, analyzed and interpreted data, performed statistical analysis, and co-wrote the manuscript. All authors read and approved the final manuscript.
